# Evaluation of aortic root for definition of prosthesis size by magnetic resonance imaging and cardiac computed tomography: implications for transcatheter aortic valve implantation

**DOI:** 10.1186/1532-429X-13-S1-P243

**Published:** 2011-02-02

**Authors:** Ralf Koos, Ertunc Altiok, Andreas Horst Mahnken, Mirja Neizel, Guido Dohmen, Nikolaus Marx, Harald Kühl, Rainer Hoffmann

**Affiliations:** 1 Department of Cardiology, University hospital RWTH Aachen, Aachen, Germany; 2 Department of Diagnostic Radiology, University hospital RWTH Aachen, Aachen, Germany; 3 University Hospital Duesseldorf, Duesseldorf, Germany; 4 Department of Cardiac Surgery, University hospital RWTH Aachen, Aachen, Germany; 5 Department of Cardiology, Klinikum Harlaching, München, Germany

## Introduction

Prior to transcatheter aortic valve implantation (TAVI), dual-source computed tomography (DSCT) is currently the preferred imaging modality for correct prothesis selection by accurate analysis of aortic annulus and ascending aorta. However, DSCT is associated with the need for contrast administration and radiation exposure.

## Purpose

This study aimed to compare cardiac magnetic resonance imaging (CMR) with dual source computed tomography (DSCT) for analysis of aortic root dimensions prior to (TAVI). In addition, the potential impact of CMR and DSCT measurements on TAVI strategy defined by 2D-transesophageal echocardiography (TEE) was evaluated.

## Methods

Aortic root dimensions were measured using CMR (1.5 Tesla, Achieva, Philips, the Netherlands) and DSCT (Definition, Siemens, Erlangen, Germany) in 58 patients referred for evaluation of TAVI. For CMR non-contrast enhanced navigator-gated 3-D whole heart acquisition was conducted (voxel size 1.2x1.2x1.8, TR/TE 4.9/2.9, flip angle 100°). CMR-images were analyzed using a 3-D reconstruction tool (EWS, Philips, the Netherlands). Coronal and sagittal CT-images were reconstructed for evaluation of aortic annulus and ascending aorta dimensions at an external workstation. The TAVI strategy (choice of prosthesis size and decision to implant) was based on 2D-TEE annulus measurements.

## Results

CMR and DSCT aortic root measurements showed an overall good correlation (r=0.86, p<0.001 for coronal aortic annulus diameters). There was also a good correlation between TEE and CMR as well as between TEE and DSCT for measurement of sagittal aortic annulus diameters (r=0.69, p<0.001). However, annulus diameters assessed by TEE (22.1 ± 2.3mm) were significantly smaller than coronal aortic annulus diameters assessed by CMR (23.4 ± 1.8mm, p<0.001) or DSCT (23.6 ± 1.8, p<0.001). Regarding TAVI strategy, the agreement between TEE and sagittal CMR (kappa = 0.89) as well as sagittal DSCT measurements (kappa = 0.87) was perfect. However, decision based on coronal CMR- or MSCT measurements would have modified TAVI strategy as compared to a TEE based choice in a significant number of patients (22% to 24%).

## Conclusions

In patients referred for TAVI, CMR measurements of aortic root dimensions show a good correlation with DSCT measurements and thus CMR may be an alternative 3D-imaging modality without the need for radiation or contrast-agent exposure. Aortic annulus measurements using TEE, CMR and DSCT were close but not identical and the method used has important potential implications on TAVI strategy.

